# Supplementary figures and images for: Elevated Hoxb5b Expands Vagal Neural Crest Pool and Blocks Enteric Neuronal Development in Zebrafish
Source: Front Cell Dev Biol. 2022 Jan 31;9:803370. doi: 10.3389/fcell.2021.803370 (PMC8841348; doi:10.3389/fcell.2021.803370)

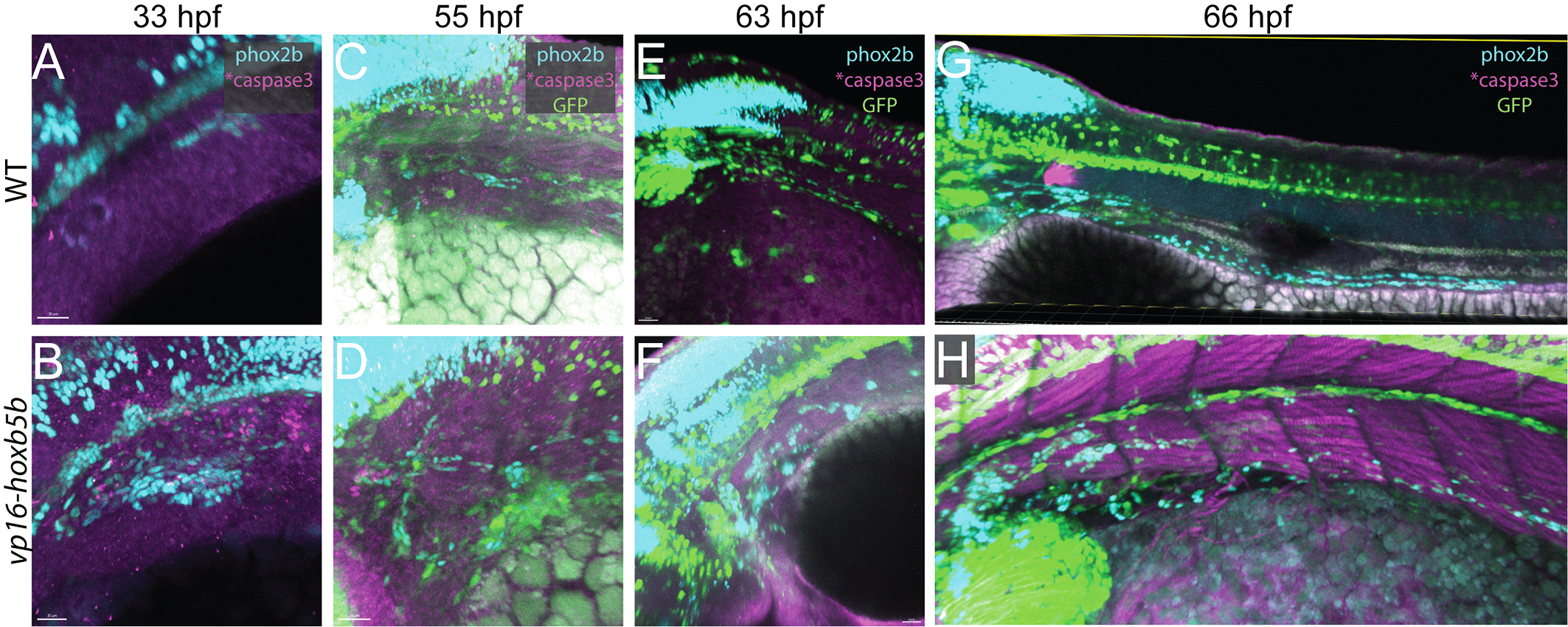

Supplement: Supplementary file 1 [file Image3.TIF]

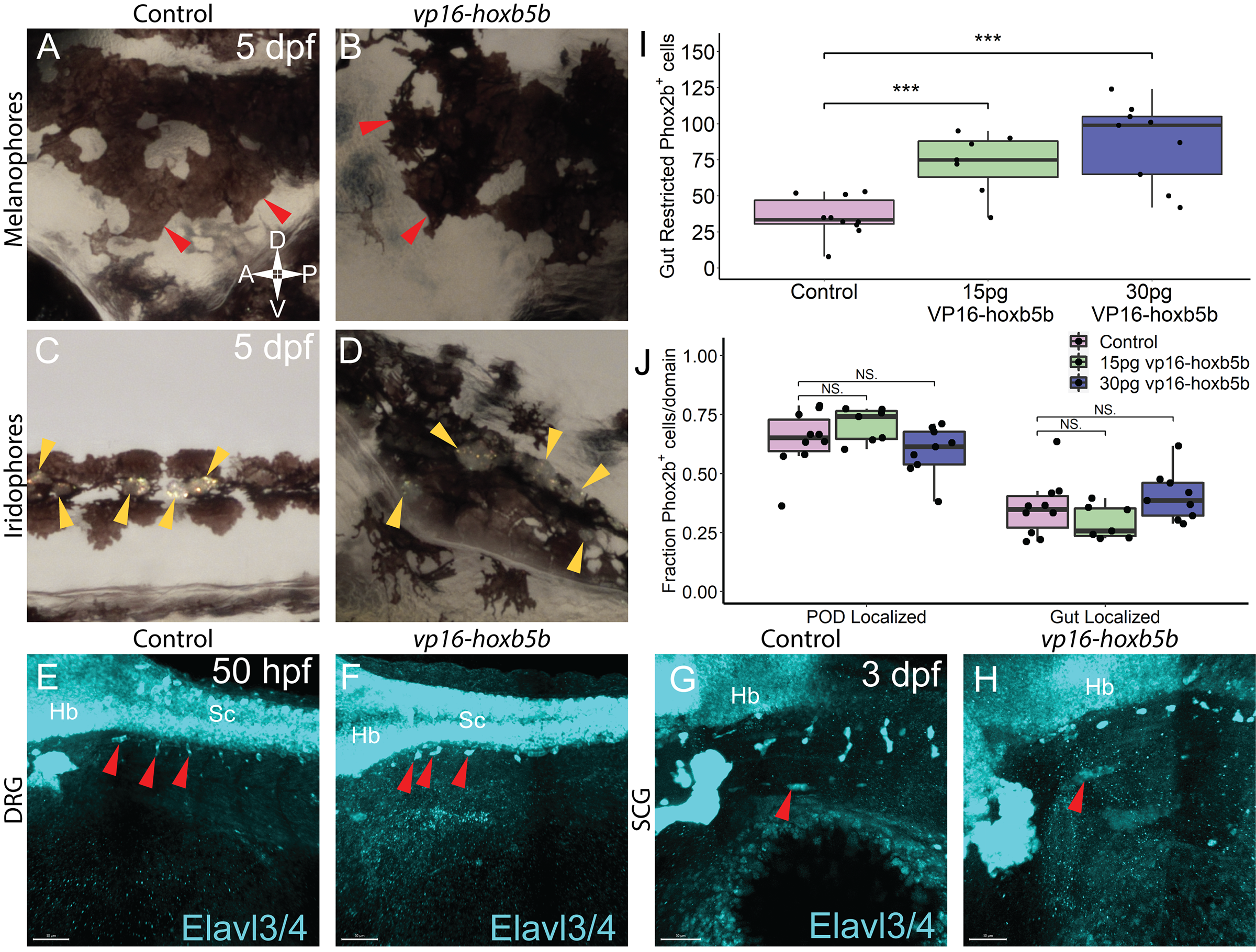

Supplement: Supplementary file 2 [file Image4.TIF]

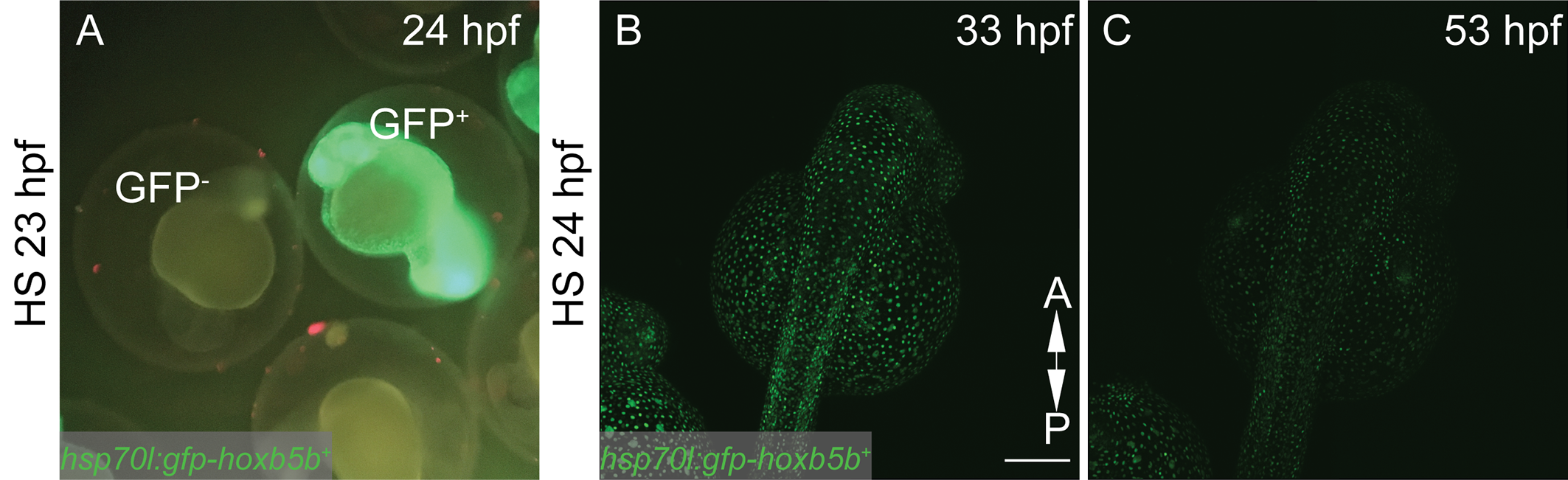

Supplement: Supplementary file 3 [file Image2.TIF]

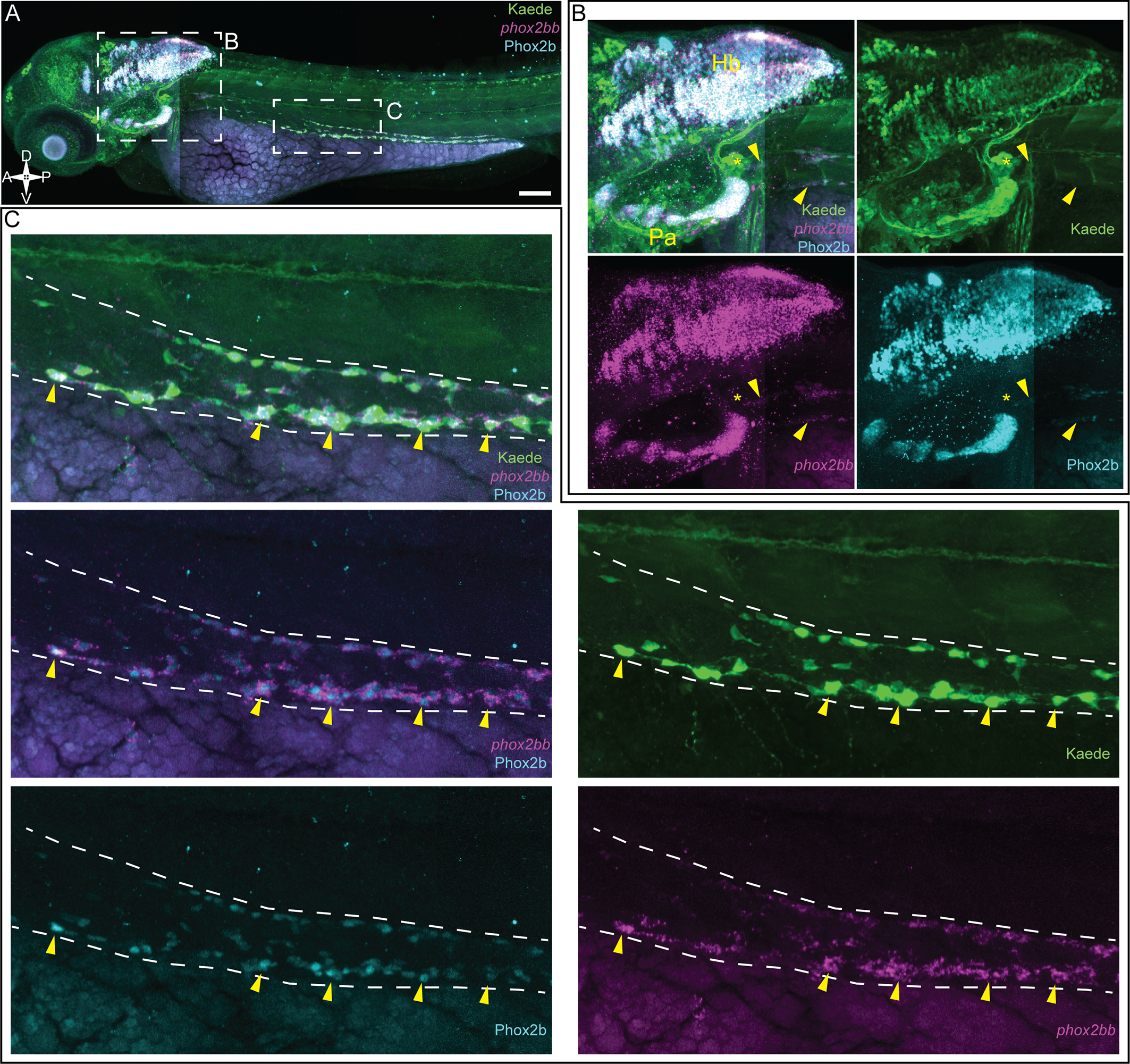

Supplement: Supplementary file 4 [file Image1.TIF]
